# Study protocol for design of a personalized dietary supplement based on the gut microbiota of Alzheimer’s patients and evaluation of its effects in a pilot randomized controlled trial

**DOI:** 10.3389/fnut.2025.1653841

**Published:** 2025-09-23

**Authors:** Sara Clemente-Velasco, Beatriz de Lucas, Mariangela Tabone, Carlo Bressa, María del Rocío González-Soltero, Sara Martínez-López, María Bailén, Diego Domínguez-Balmaseda, Nazareth Castellanos, Gustavo G. Diez, Fuensanta Noguera-Perea, Juan Marín-Muñoz, Pilar Sánchez-Alonso, Ana Isabel Rey, Beatriz G. Gálvez, Mar Larrosa

**Affiliations:** ^1^Masmicrobiota Research Group, Madrid, Spain; ^2^Department of Food Science and Nutrition, Faculty of Pharmacy, Universidad Complutense de Madrid, Madrid, Spain; ^3^Department of Bioscience, Faculty of Biomedical and Health Sciences, Universidad Europea de Madrid, Madrid, Spain; ^4^Faculty of Sport Sciences, Universidad Europea de Madrid, Madrid, Spain; ^5^Facultad de Ciencias Experimentales, Universidad Francisco de Vitoria, Madrid, Spain; ^6^Hospital La Paz Institute for Health Research – IdiPAZ, Madrid, Spain; ^7^Department of Preventive Medicine, Public Health and Microbiology, Faculty of Medicine, Universidad Autónoma de Madrid, Madrid, Spain; ^8^Real Madrid Graduate School, Faculty of Sports Sciences, Universidad Europea de Madrid, Madrid, Spain; ^9^Nirakara Lab, Complutense University of Madrid, Madrid, Spain; ^10^Unidad de Demencias, Hospital Clínico Universitario Virgen de la Arrixaca, Murcia, Spain; ^11^Unidad de Enfermedades Neurodegenerativas y Trastornos del Movimiento, Hospital Universitario Puerta de Hierro, Majadahonda, Spain; ^12^Animal Nutrition, Department of Animal Production, Faculty of Veterinary, Universidad Complutense de Madrid, Madrid, Spain; ^13^Department of Biochemistry and Molecular Biology, Faculty of Pharmacy, Universidad Complutense de Madrid, Madrid, Spain

**Keywords:** precision nutrition, artificial intelligence, microbiota, gut-brain axis, short-chain fatty acids

## Abstract

**Background:**

Increasing evidence links gut microbiota composition to neurological disorders, including Alzheimer’s disease (AD), through the gut-brain axis. Microbial metabolites such as lipopolysaccharide (LPS) and butyrate are associated with AD progression. Among modifiable factors, diet plays a central role in shaping gut microbiota and may influence disease-related microbial patterns. Personalized nutrition based on individual microbiota profiles offers a promising strategy to modulate these biomarkers. This protocol describes a study designed to develop a personalized dietary supplement for patients with AD by integrating microbiota, clinical, and dietary data using artificial intelligence (AI) and network analysis. A secondary objective is to assess in a pilot study the short-term effects of the supplement on microbiota composition, function, and plasma metabolomics to identify modifiable biomarkers.

**Design:**

This two-phase study will begin with baseline data collection from 60 patients with AD and 60 healthy controls. AI and network-based analyses will identify dietary and microbial variables predictive of disease status. Based on these findings, a personalized supplement will be formulated using dietary components—such as fibers, polyphenols, or fatty acids—targeting microbial taxa and metabolic pathways associated with AD. In the second phase, 60 patients with AD will be randomized (1:1) to receive either the personalized supplement or a standard product for 3 months. Effects on microbial taxa, LPS, short-chain fatty acids (SCFAs), and plasma metabolites will be evaluated before and after the intervention.

**Methods:**

Participants will undergo assessments of lifestyle factors, including diet and physical activity, as well as measurements of blood LPS and fecal SCFAs. Machine learning and network analyses will explore links among microbiota, diet, and clinical features. Predictive variables will guide supplement design. The intervention will evaluate changes in LPS, butyrate, and microbial markers. Plasma metabolomic profiles will also be analyzed. Data integration using AI and network approaches will help identify biomarkers and assess intervention outcomes.

**Discussion:**

Combining AI and network analysis in microbiota research supports the development of personalized nutrition strategies in AD. This approach may modulate disease-related microbiota and systemic markers, contributing to innovative therapeutic tools and advancing both Alzheimer’s care and nutritional science.

**Clinical trial registration:**

ClinicalTrials.gov, identifier NCT06199193.

## 1 Introduction

Alzheimer’s disease (AD) is an irreversible degeneration of the brain that causes disorders of memory, cognition, personality, and other functions, ultimately leading to death due to complete cessation of brain activity. It is estimated that there are 50 million people worldwide living with dementia, and two-thirds of them suffer from AD. By 2030, without new discoveries, this number is expected to rise to nearly 75 million patients (1). The gut microbiota plays a crucial role in the bidirectional communication between the gut and the brain, and accumulating evidence suggests its involvement in AD pathophysiology (2). For instance, elevated levels of bacterial lipopolysaccharides (LPS) have been positively associated with cerebral amyloidosis, while higher production of short-chain fatty acids (SCFAs), particularly butyrate, by the gut microbiota has been linked to reduced amyloid deposition (3).

Currently, there is no effective treatment for AD, but studies indicate that following a healthy diet, such as the Mediterranean diet (MD), and engaging in physical activity may delay the symptoms of the disease (4, 5). Adherence to Mediterranean and Mediterranean-DASH Intervention for Neurodegenerative Delay (MIND) diets has been associated with reduced risk of cognitive decline in observational studies (6, 7). However, the first randomized controlled trial of the MIND diet (8) did not show significant differences in cognitive outcomes compared to a healthy control diet. Recent meta-analyses continue to support potential protective effects of Mediterranean-type diets but emphasize heterogeneity across populations and study designs (9). Nutritional supplementation has also been widely investigated. A recent meta-analysis of omega-3 fatty acids, docosahexaenoic acid (DHA), eicosapentaenoic acid (EPA), and Souvenaid^®^ concluded that these interventions may modestly attenuate cognitive decline, with benefits most consistently observed in Clinical Dementia Rating scores, though overall findings remain heterogeneous and not statistically conclusive (10). Evidence from randomized controlled trials indicates that physical exercise is the most consistently beneficial lifestyle intervention, improving quality of life, mobility, and certain cognitive domains, even though effects on biomarkers remain inconclusive (11). Combined interventions, particularly those integrating exercise with cognitive stimulation or other modalities, appear promising but yield heterogeneous results and require further validation (11). Overall, while observational studies consistently suggest protective effects of diet and lifestyle factors on AD onset, randomized trials in patients indicate only modest benefits on disease progression, particularly with nutritional supplements and exercise.

Since many of these lifestyle and nutritional interventions are known to modulate the gut microbiota, it has been suggested that targeting microbial composition and activity through diet could represent a novel or adjuvant therapeutic strategy in AD (12). In this regard, foods or dietary patterns that reinforce the intestinal barrier to reduce the translocation of LPS into the bloodstream (and ultimately to the brain), and/or reduce populations of Gram-negative bacteria (carriers of LPS), and/or increase butyrate production by the microbiota, could serve as adjuvant treatments for patients with AD (13). However, these patients are often highly resistant to lifestyle changes, showing poor adherence to new dietary habits, and the responsibility for implementing these changes often falls on caregivers, increasing their physical and emotional burden (14). For these reasons, the use of dietary supplements providing essential and beneficial components of the diet could be an effective strategy as an adjuvant treatment for these individuals (15).

There is increasing evidence that physiological responses to diets or dietary components depend on individual characteristics (sex, genetics, age), dietary habits, and, in part, on the composition of the gut microbiota (16, 17). Therefore, for dietary interventions to be successful, they must be adapted by considering various individual factors such as dietary habits, personal characteristics, and microbiota composition (18). To study the interactions among individual characteristics, microbiota composition, and diet, and to integrate all this data to understand changes in human physiology, new computational approaches such as artificial intelligence (AI) are increasingly being used (19). These methods help us understand complex interactions, treating the individual as a system in order to predict physiological changes resulting from external modifications such as diet. In fact, some of these computational methods, including machine learning (ML) algorithms, have already been used to design personalized diets and improve health-related parameters (16, 20, 21). By combining these concepts, personalized microbiota-based nutrition, along with computational tools such as ML and network analysis, is beginning to be used to predict clinical phenotypes, opening the door to new advances in the prevention and treatment of diseases through this approach (18). Although most of our current knowledge comes from experimental studies, it should be possible to develop mechanistic-based mathematical models to assist in designing intervention strategies (22).

However, despite increasing evidence linking gut microbiota, diet, and AD, there are currently no personalized dietary supplements specifically designed for patients with AD that integrate individual clinical, dietary, and microbiota data. Existing nutritional interventions are largely generic and do not account for patient heterogeneity or the mechanistic pathways connecting diet, microbiota, and AD-related biomarkers. This gap highlights the need for the present protocol, which aims to address these limitations by proposing a data-driven framework for supplement development and preliminary validation.

This protocol describes a study whose aim is to investigate the feasibility of designing personalized nutritional supplements for patients with AD based on their sex, age, medical history, dietary habits, physical activity, and gut microbiota, applying AI and bioinformatics tools through a data-driven approach. In this context, study success, referred to as “greater efficacy” is defined as promoting a microbiota with a higher presence of health-associated species, reducing microbial taxa related to AD, and modifying AD-related biomarkers. The use of a combination of predictive and causal approaches (including network analysis) to integrate and simultaneously analyze patient clinical data, dietary and exercise habits, and microbiota profiles will allow us to identify dietary variables that should be modified to induce changes in factors related to AD prevention (e.g., butyrate) or progression (e.g., LPS and microbiota markers). The overarching goal is to determine whether such a tailored supplement would exhibit greater efficacy than currently available products. A medical food product designed to support brain health in individuals with AD will be used as a comparator. Finally, the study aims to identify new AD-related markers modified by the designed supplement using a metabolomic approach.

The findings may lead to the development of new, customized nutritional products by food and pharmaceutical companies, offering substantial socio-economic benefits. Moreover, the study’s multidisciplinary approach could advance both Alzheimer’s care and the science of personalized nutrition, providing a model for future research and clinical applications.

## 2 Methods

### 2.1 Participants

Patients diagnosed with AD at the Dementia Unit of “Hospital Clínico Universitario Virgen de la Arrixaca” (HCUVA) in Murcia and the Neurodegenerative Diseases and Movement Disorders Unit of “Hospital Universitario Puerta de Hierro” (HUPHM) in Madrid (Spain), who meet the inclusion criteria and none of the exclusion criteria, and who are willing to participate in the study, will be recruited. Caregivers may also participate as control subjects. Recruitment will be conducted by neurologists from the research team. Potential participants will be identified among current patients of the Dementia Unit and the Neurodegenerative Diseases Unit. Participants will be recruited from HCUVA and HUPHM. Inclusion criteria will be men and women aged 60 to 85 years with AD at Global Deterioration Scale (GDS) stage 3, confirmed by cerebrospinal fluid (CSF) biomarkers. Availability of a study partner who has regular contact with the participant, can accompany them to study visits as needed, assists with supplement intake and study procedures, and can provide collateral information. Healthy control group, healthy caregivers of patients with AD may be included as control subjects if they meet the eligibility criteria. Exclusion criteria include the presence of other diseases that may interfere with sample results, such as gastrointestinal disorders (e.g., ulcerative colitis, Crohn’s disease), autoimmune diseases, chronic antibiotic use or antibiotic treatment within the past 3 months, chronic treatment with immunosuppressants, corticosteroids or immunomodulators, a history of cancer treated with chemotherapy in the past 5 years, intellectual disability, or acquired brain injury.

### 2.2 Interventions

**Figure 1:**
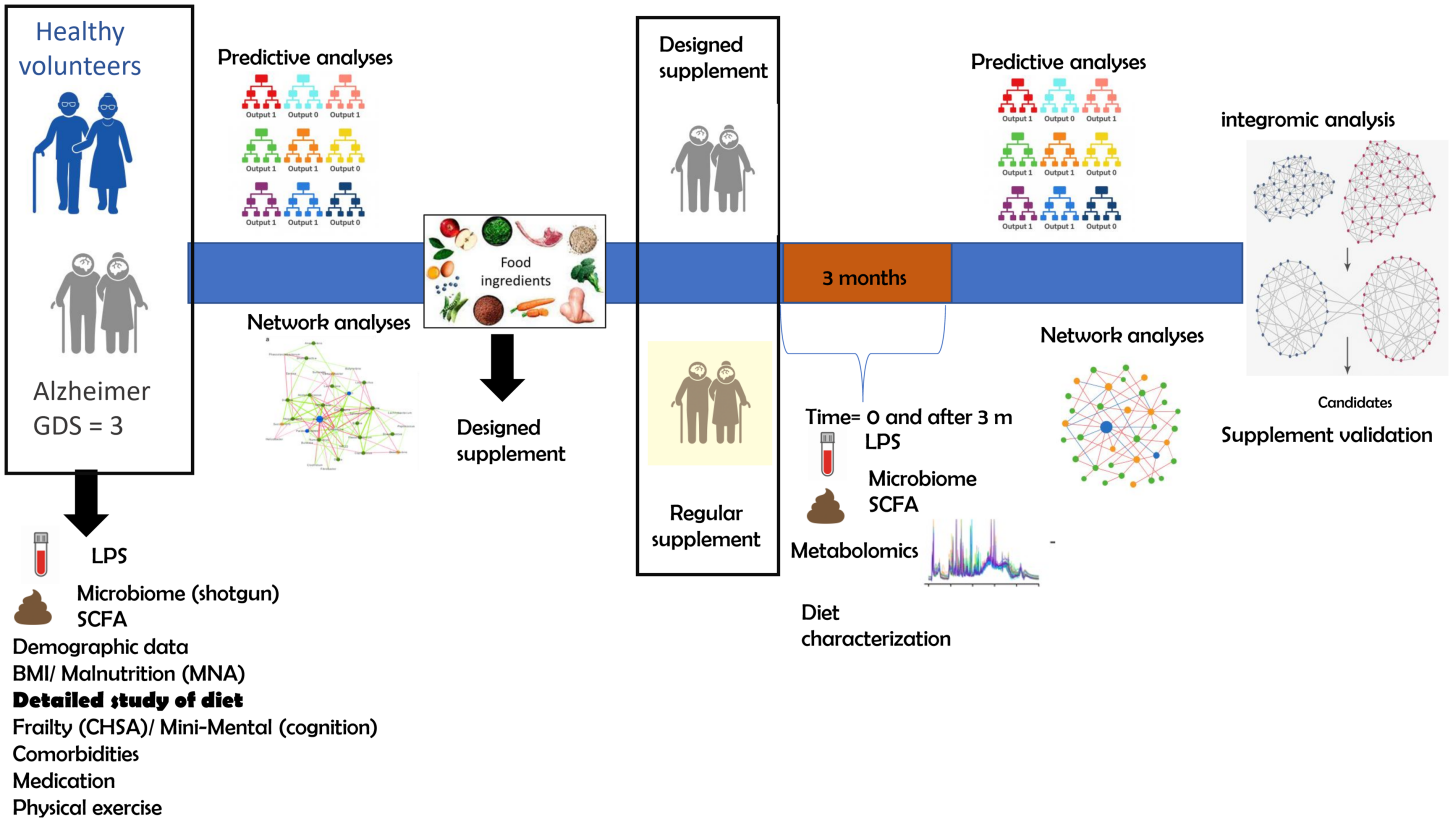
Schematic representation of the study.

The study consists of three main stages (Figure 1). At baseline (*T* = 0), both healthy subjects and patients will undergo characterization, including demographic data, physical characteristics (age, sex, BMI, etc.), frailty (Clinical Frailty Scale for Accumulation of Deficits, CFS), malnutrition (Mini Nutritional Assessment questionnaire, MNA), clinical data (biochemistry, hematology, apolipoprotein E (APOE) genotyping, inflammatory markers, etc.), diet, physical activity (International Physical Activity Questionnaire for the Elderly, IPAQ-E), gut microbiota (diversity, composition, and community interactions), blood LPS and butyrate levels, as well as comorbidities and medications. All the collected variables will be subjected to network and predictive analyses (e.g., random forest) to examine interactions between variables and identify modifiable predictors of AD through dietary intervention. In the second stage, a personalized supplement will be designed based on the characteristics of patients with AD, though patients themselves will not be involved in this phase. In the third stage, a randomized, parallel-group nutritional intervention trial will be conducted to compare the “*ad hoc*” designed supplement with a standard nutritional supplement commonly used in patients with AD. The interval between baseline characterization (*T* = 0) and the beginning of the intervention (*T* = 2) will vary depending on recruitment, sequencing, and data processing times. The intervention will start as soon as possible after completion of these analyses, in order to maximize the relevance of the baseline analyses. During this period, participants will continue their usual medical care without changes. The intervention will last 3 months (pre-intervention: *T* = 2; post-intervention: *T* = 3) to evaluate the supplement’s effectiveness in modulating the microbiota, LPS levels, and butyrate production. Additionally, a metabolomic analysis will be conducted to identify new biomarkers related to the supplement’s effects on the disease.

A participant in the study may withdraw their consent at any time, without the need to provide any justification. This decision will not entail any liability or negative consequences for the participant. Individuals who choose to withdraw will not undergo further follow-up and will not be replaced. The investigator reserves the right to withdraw a participant from the study if it is determined that the participant can no longer comply with the study requirements or if any of the procedures are deemed potentially harmful to the participant’s wellbeing. Any data collected from participants prior to withdrawal will be retained and included in the analysis; however, no additional data will be collected after the point of withdrawal. Participants may be excluded or withdrawn from the study, even after sample collection, if they meet any of the following criteria:

- Occurrence of an adverse event that, in the investigator’s judgment, warrants withdrawal.
- Protocol deviation that compromises the interpretation or scientific validity of the study results.
- Medical decision.
- Voluntary decision by the participant to discontinue participation.
- Loss to follow-up.

During the sample collection period, participants will continue attending their regular medical visits and receiving standard clinical follow-up.

To monitor compliance with the intervention, participants, with the assistance of their study partner, will complete a supplement intake tracker. This will consist of a daily diary (paper-based) where supplement intake, timing, and any missed doses will be recorded. Returned bottles/sachets will also be counted at each follow-up visit, and weekly phone calls will be conducted to reinforce adherence and address potential barriers. Adherence will be calculated as the percentage of prescribed doses consumed. Participants with ≥80% adherence will be considered compliant.

### 2.3 Outcomes

The primary outcome of this study is to detect differences in gut microbiota diversity, composition, and functionality between healthy controls and AD participants 1.5 years after baseline (*T* = 0), and to investigate changes in gut microbiota in AD participants before and after the 3-month nutritional intervention. Microbiota analysis will be performed on fecal samples using shotgun metagenomic sequencing. Secondary outcomes include the characterization of dietary habits in both healthy controls and AD participants at baseline (*T* = 0), using a 3-day food record, a food frequency questionnaire (FFQ), and a Mediterranean diet (MD) adherence screener (MEDAS). Physical activity will be assessed using the International Physical Activity Questionnaire for the Elderly (IPAQ-E). Blood lipopolysaccharide (LPS) levels will be measured at baseline (*T* = 0), before (*T* = 2), and after (*T* = 3) the 3-month intervention using the Limulus amebocyte lysate (LAL) method. Fecal short-chain fatty acids (SCFAs) and fecal bile acids will also be measured at these timepoints using high-performance liquid chromatography with diode array detection (HPLC-DAD). To explore other variables potentially modified by the nutritional intervention, an untargeted metabolomic analysis will be conducted on blood samples using liquid chromatography coupled to high-resolution mass spectrometry (LC-HRMS). Variables and the corresponding assessment tools are summarized in Table 1.

**Table 1:** Summary of assessments and testing instruments used in the study.

Variables	Instruments/assessment tools
Health and sociodemographics	Data collected by the Data Monitoring Committee (DMC): socio-demographic information, household income, education level, animal contact, smoking status.
Clinical variables	Age at diagnosis, duration of symptoms, GDS stage, CSF biomarkers, pharmacological treatments
Physical activity	International Physical Activity Questionnaire for Elderly (IPAQ-E) Clinical Frailty Scale (CFS)
Biological samples	
Blood (plasma)	LPS: Limulus amebocyte lysate (LAL) method; Untargeted metabolomics (LC-HRMS)
Stool	SCFAs and bile acids: HPLC-DAD; Gut microbiota: WGS, network analysis, random forest analysis
Nutritional status	
Dietary habits	Food Frequency Questionnaire (FFQ); 3-day food record; Mediterranean diet adherence questionnaire (MEDAS)
Neuropsychological assessment	
Global cognitive function	Mini Mental Examination test (MMSE)
Stress assessment	
	Perceived Stress Scale (PSS-10)

### 2.4 Sample size calculation

To calculate the sample size, the method used to test the association between the abundance of a microbiota taxon and exposure to a variable or group of variables was applied (23). The bacterial genus *Odoribacter* was selected for this purpose, as its abundance has shown a significant role in predicting AD (24). The mean and standard deviation of the relative abundance of this genus were 0.26 ± 0.42 in healthy individuals and 0.77 ± 0.77 in patients with AD, according to data obtained directly from the authors of Liang et al. (25). The pooled standard deviation was 0.6533, and the calculated effect size was: (0.77–0.26)/0.6533 = 0.7806. Considering this effect size, and assuming a significance level (*α*) of 0.05 and statistical power (1 − *β*) of 0.80, the formula used for calculating the sample size was: n=[2×(z₁⋅α/2+z₁⋅β)2]/(effect size)2 . Substituting the values yields a required sample size of 25 individuals per group. To account for an estimated 20% dropout rate, the final sample size was adjusted to 30 participants per intervention group. Accordingly, the cross-sectional stage (stage 1) will be conducted with *n* = 60 participants per group, which will provide robustness to detect differences between healthy individuals and patients with AD. In the subsequent intervention phase, which will be carried out exclusively in patients with AD, these 60 individuals will be randomized into two arms: one receiving the designed supplement and the other a standard supplement. The aims of this pilot intervention are to: (i) estimate trends and effect sizes of the intervention on the microbiota and clinical parameters, (ii) evaluate the feasibility and acceptability of the intervention protocol in this vulnerable population, and (iii) collect preliminary data to power a future definitive randomized controlled trial. The intervention will last 3 months, as the aim is to obtain preliminary results in a pilot context.

### 2.5 Allocation

For the nutritional intervention study, patients with AD will be randomly assigned to two groups (*n* = 30 each) using the free randomization software OxMar (26). Randomization will not be stratified by study site. However, the study site will be included as a covariate in the statistical models to control for its potential effect as a confounding variable. The control group will receive a standard nutritional supplement commonly used in patients with AD (Souvenaid^®^, Nutricia, Madrid, Spain), and dietary recommendations will be provided if their usual intake does not meet the established nutritional goals for this population. The intervention group will receive the newly designed supplement, along with tailored dietary recommendations. The group assignment will be performed by the team of neurologists. Supplement blinding in terms of format, flavor, odor, etc., is not planned in this study. Supplements will be labeled as either “A” or “B,” and participants will be informed that they are taking a supplement but will not know whether it is the standard or the “*ad hoc*” formulation. To eliminate bias, the researchers conducting the experiments and performing the analyses will remain blinded to group allocation. Baseline demographic and clinical information will be collected for participants who discontinue the study, along with available data on health status, adverse events, and primary efficacy outcomes.

### 2.6 Data management

All study variables will be managed using Research Electronic Data Capture (REDCap) software (27, 28) with each participant assigned a code to ensure anonymity. The database will include all demographic and clinical variables required for the study. The first 10 data entries will be double-checked by co-investigators, a process that will also serve as additional training for data collectors. All relevant data concerning study participants and the associated measurements will be safeguarded by the principal investigator (PI), who will ensure the confidentiality and anonymity of the data at all times. All documentation related to the study will be stored in the Investigator’s File at each participating center under the custody of the PI until study completion. Once the study concludes, all documents will be indexed and transferred to the general archive of the center, following Good Clinical Practice (GCP) guidelines. The PI will ensure that subject identification codes are preserved for at least 15 years after the study’s conclusion or interruption. In all cases, the PI will guarantee the confidentiality of the data and the documents stored in the archive.

### 2.7 Sociodemographic and lifestyle data

Participants’ demographic information, including age, sex, and ethnicity, as well as socioeconomic factors such as household income, number of household members, and education level will be collected. Lifestyle habits, including alcohol consumption, smoking, and contact with animals, will also be recorded. Clinical variables such as time since disease onset, age at diagnosis, and the affected brain regions will be documented, along with cerebrospinal fluid (CSF) biomarkers such as Abeta42 and phosphorylated Tau levels. Behavioral symptoms and pharmacological treatments (e.g., cholinesterase inhibitors, memantine, neuroleptics, and trazodone) will also be assessed. To evaluate dietary habits in both patients and healthy controls, a comprehensive dietary assessment will be conducted. The following validated tools will be used: a food frequency questionnaire (FFQ) (29) assessing habitual intake over the past year, a 3-day food record collected during the same week as microbiota sampling, and the 14-item Mediterranean Diet Adherence Screener (MEDAS) (30). Food intake data will be analyzed using DIAL software (version 3.15.0.0) (31) to determine intake of macronutrients, micronutrients, fiber, and other bioactive compounds, including: complex carbohydrates and simple sugars, animal and plant-based proteins, saturated, monounsaturated, and polyunsaturated fats, soluble and insoluble fiber, ethanol, and water- and fat-soluble vitamins. Physical activity will be assessed using the International Physical Activity Questionnaire for the Elderly (IPAQ-E) (32) and the Clinical Frailty Scale (CFS) (33).

### 2.8 Neuropsychological assessment

To assess global cognitive function, neurologists will administer the Mini-Mental State Examination (MMSE) (34). This test evaluates several cognitive domains, including orientation, registration, attention and calculation, recall, language, and visuospatial abilities.

### 2.9 Assessment of perceived stress

Participants’ perceived stress levels will be assessed using the validated 10-item Perceived Stress Scale (PSS-10) (35, 36). The PSS-10 measures the degree to which individuals perceive their lives as unpredictable, uncontrollable, and overloaded during the past month. Items are rated on a 5-point Likert scale from 0 (“never”) to 4 (“very often”), and scores are summed to provide a total perceived stress score, with higher scores indicating greater perceived stress.

### 2.10 Monitoring

The Data Monitoring Committee (DMC) is composed of a multidisciplinary team including seven neurologists and five neuropsychologists. Neurologists play a central role in participant selection and in ongoing monitoring throughout the study. To identify any emerging safety concerns during the nutritional intervention, such as adverse effects, interim analyses will be conducted and may lead to protocol modifications or early termination of the trial if necessary. Interim analyses will be performed weekly during the 3-month intervention period by two members of the research team. In addition, participants will be provided with a dedicated telephone number to report any adverse events, particularly those related to gastrointestinal symptoms. This direct communication channel will ensure continued participant engagement and allow for timely detection and documentation of adverse events. The monitoring plan includes both solicited and spontaneously reported events and ensures proper documentation, evaluation, and reporting of any unintended effects arising from the study intervention.

### 2.11 Biological samples

A total of 10 mL of blood will be collected in EDTA tubes by a hospital nurse and processed in hospital biobanks registered in the Spanish National Biobank Registry under reference numbers PT17/0015/0038 (HCUVA) and B0000920 (HUPHM). For stool samples, participants will be provided with materials to collect three samples (one per week for three consecutive weeks), along with an appointment for delivery. Participants will receive detailed instructions on how to collect and store the samples, including a simple, illustrated information booklet and an explanatory video. Stool collection will use Fe-Col^®^ feces collection paper (Alpha Laboratories, Hampshire, United Kingdom) and the Danastool kit (Danagen-Bioted, Badalona, Spain), which includes a tube with an integrated spoon, a participant identification label, and 8 mL of fecal DNA stabilizing reagent. This system maintains sample stability for several months at room temperature (15–25°C) and indefinitely at −20 or −80°C, allowing home collection and convenient hospital delivery. The date of sample collection and delivery will be recorded. Once in the laboratory, stool samples will be processed and stored at −80°C. One aliquot will be deposited in the hospital biobank. Stool consistency will be self-classified by participants using the Bristol Stool Scale.

### 2.12 Microbiota analysis

For fecal microbiota analysis, DNA will be extracted using the Danagene Microbiome Fecal DNA kit (Danagen-Bioted, Badalona, Spain). DNA concentration will be measured using the Quant-iT™ PicoGreen^®^ dsDNA Assay Kit (Invitrogen, Waltham, MA, United States).

### 2.13 Whole genome sequencing

Shotgun metagenomic sequencing will be performed on stool samples to determine microbiome composition and function. For library preparation, DNA will be randomly sheared into short fragments. The resulting fragments will undergo end-repair, A-tailing, and ligation with Illumina adapters. Adapter-ligated fragments will be size-selected, PCR-amplified, and purified. Library quality will be assessed using a Quantus™ Fluorometer (Promega, Madison, WI, United States) for quantification, and an Agilent Bioanalyzer (Santa Clara, CA, United States) for size distribution. Pooled libraries will be sequenced on the Illumina NovaSeq 6,000 platform. Raw sequencing data will be processed using the bioBakery Whole Metagenome Shotgun (wmgx) workflow, publicly available at http://huttenhower.sph.harvard.edu/biobakery (37). Quality control will be performed using the KneadData tool (v0.12.0), which includes trimming and decontamination steps. Trimming will be conducted with Trimmomatic (v0.39), using default parameters (SLIDINGWINDOW:4:20 MINLEN:50) to remove adapters, low-quality reads, and short sequences. Host-derived contaminants will be removed with Bowtie2 (v2.5.1) by aligning reads to the hg37_and_human_contamination reference database using the “very sensitive” preset, as recommended in the KneadData documentation (38, 39). Species-level microbial profiling (including bacteria, archaea, eukaryotes, and viruses) will be performed with MetaPhlAn (v4.1) using the latest database (mpa_vJun23_CHOCOPhlAnSGB_202403) (40). Functional profiling of microbial metabolic pathways and other molecular functions will be performed using HUMAnN (v3.0) (41) with the following databases: full_chocophlan.v201901_v31, uniref50_annotated_v201901b_full, uniref90_annotated_v201901b_full, uniref50_ec_filtered_201901b_subset, uniref90_ec_filtered_201901b_subset, and full_mapping_v201901b.

### 2.14 Measurement of plasma lipopolysaccharide

Plasma LPS levels, used as a marker of intestinal permeability and systemic endotoxin exposure, will be determined using the Pierce™ LAL Chromogenic Endotoxin Quantitation Kit (Thermo Fisher Scientific, Madrid, Spain).

### 2.15 Short-chain fatty acids

To determine SCFAs in human feces, we will employ the method described by Wang et al. (42) with some modifications. Fecal samples (200 mg) will be homogenized with 2 mL of 30% aqueous acetonitrile (ACN) and vortexed for 5 min to extract the SCFAs. The suspension will be filtered and centrifuged at 4,000 × g at 4°C for 10 min. Chemical derivatization of SCFAs will be performed by mixing 0.4 mL of the sample supernatant with 0.2 mL of 200 mM 3-nitrophenylhydrazine (NPH) solution and 0.2 mL of a mixture containing 120 mM N-(3-dimethylaminopropyl)-N′-ethylcarbodiimide and 6% pyridine solution, in borosilicate test tubes. The mixture will then be incubated at 40°C for 45 min. An HPLC system (Agilent 1,100) equipped with a quaternary gradient pump, an online degasser, an autosampler, a thermostatically controlled column compartment, and a UV–Vis photodiode array detector will be used. Sample separation will be carried out on an Agilent LiChroCART 150 × 4.6 mm Purospher STAR RP-18e column (5 μm particle size) at a constant flow rate of 0.6 mL/min and a column temperature of 30°C. The mobile phase will consist of water (A) and acetonitrile (B), with gradient elution as follows: 10–40% B from 0 to 15 min, and 40–80% B at 30 min.

### 2.16 Fecal bile acids

Bile acids in feces will be analyzed following the method described by Kakiyama et al. (43). Lyophilized stool samples will be suspended in water and heated at 90°C for 10 min. The samples will then undergo ultrasonication, followed by the addition of sodium acetate buffer (100 mM, pH 5.6) containing 15 units of cholylglycine hydrolase and 150 units of sulfatase. This mixture will be incubated at 37°C for 16 h. To terminate the reaction, isopropanol will be added, and the mixture will be reheated at 90°C for 10 min. An internal standard, 50 nmol of nor-deoxycholic acid (norDCA), along with 0.1 N NaOH, will then be added to the mixture. Bile acids will be extracted from the fecal matrix by ultrasonication at room temperature for 1 h. After centrifugation, the supernatant will be transferred to a glass test tube, and the pellet will be washed with 0.1 N NaOH. The combined extract will be applied to a Waters Sep-Pak tC18 cartridge, from which retained bile acids will be eluted with methanol and evaporated to dryness under a nitrogen stream. The extracted unconjugated bile acids will be derivatized to their 24-phenacyl esters by adding 10 mg/mL of triethylamine (TEA) in acetone and 12 mg/mL of phenacyl bromide (2-acetobromophenone) in acetone to the dried extract. This mixture will be heated at 50°C with ultrasonication for 1.5 h. The reaction mixture will be diluted with acetone and applied to a Waters Sep-Pak^®^ silica cartridge. To elute the bile acid 24-phenacyl esters, the column will be washed with acetone, and the collected effluent will be dried under a nitrogen stream. The residue will be resuspended in 82% methanol, filtered through a 0.45 μm filter, and an aliquot will be injected into an HPLC system (Agilent 1,100) equipped with a quaternary gradient pump, an online degasser, an autosampler, a thermostatically controlled column compartment, and a UV–Vis photodiode array detector. A Waters Nova-Pak C18 column (300 × 3.9 mm inner diameter, 4 μm particle size), fitted with a guard column (20 × 3.9 mm inner diameter), will be used for separation and maintained at 32°C. Methanol (82%) will serve as the mobile phase at a constant flow rate of 0.65 mL/min. Individual bile acid 24-phenacyl esters will be detected by monitoring their absorption at 254 nm.

### 2.17 Plasma metabolome

Untargeted metabolomics in plasma will be performed using LC-HRMS, applying two complementary chromatographic methods: reversed-phase chromatography (C18 column) for hydrophobic metabolites and hydrophilic interaction chromatography (HILIC) for polar metabolites, following the protocol described by Tabone et al. (44). The LC-HRMS experiments will be conducted on an Ultimate 3,000 chromatographic system (Thermo Fisher Scientific, Courtaboeuf, France) coupled to an Exactive mass spectrometer (Thermo Fisher Scientific). Samples will be mixed with 200 μL of methanol and incubated on ice to precipitate proteins. After centrifugation, the supernatants will be collected and evaporated to dryness under a nitrogen stream using a Turbovap (Caliper Life Science Inc., Roissy, France). The resulting dried extracts will be stored at −80°C until analysis. Dried aliquots will be resuspended in 100 μL of water/acetonitrile (95:5, v/v) with 0.1% formic acid for C18 analysis, or in 100 μL of a mixture of 10 mM ammonium carbonate buffer (pH 10.5) and acetonitrile (40:60, v/v) for ZIC-pHILIC analysis. The tubes will be vortexed, sonicated in an ultrasonic bath for 5 min, and centrifuged again for 10 min. Then, 95 μL of the supernatant will be transferred to 0.2 mL vials and mixed with 5 μL of an internal standard mixture to monitor analytical consistency. Compound separation will be performed using a Hypersil GOLD C18 column (1.9 μm, 2.1 × 150 mm) maintained at 30°C (Thermo Fisher Scientific) or a Sequant ZIC-pHILIC column (5 μm, 2.1 × 150 mm) maintained at 15°C (Merck, Darmstadt, Germany). Metabolomic data processing, metabolite annotation, and pathway analysis will be performed using the Workflow4Metabolomics (W4M) platform. Metabolite annotation will rely on a spectral database based on accurately measured masses and chromatographic retention times and will be confirmed by additional LC–MS/MS experiments. The identified metabolites will be imported into the MetaboAnalyst 4.0 platform for pathway enrichment analysis.

### 2.18 Machine learning analysis to predict markers of microbiota

A classification analysis using Random Forest (RF) will be conducted to identify microbiota components associated with AD. For RF classification, we will build 3,000 decision trees. This procedure will be repeated in 30 iterations, using 100 different random seeds. Microbial taxa will be classified based on the mean prediction error across the 30 iterations. Discriminatory variables that are considered important in at least 90% of the 100 seed iterations will be identified (24). Predictor variables will be ranked based on their mean variable importance (e.g., mean decrease in accuracy) across the 30 × 100 random trials. This analysis will allow us to identify and categorize microbiota classifiers for AD in our study population, as previously described in a similarly sized Alzheimer’s patient cohort (24). In parallel, a regression-based gradient boosting model (45) will be developed to predict LPS and butyrate levels, as well as total SCFA content, based on clinical data, diet, physical activity, and microbiota profiles. This model will aggregate 1,000 of decision trees to generate predictions. Trees will be built sequentially, with each tree trained on the residuals from all previous trees, contributing incrementally to the final prediction. Features for each tree will be selected from the full dataset, including variables such as energy intake, macronutrients, micronutrients, and microbiota characteristics.

### 2.19 Network interaction analysis

To transform data such as microbial taxon abundance, dietary components, physical activity, clinical data, and LPS and butyrate levels, into interactions in a two-dimensional space, recently published and validated artificial intelligence-based methods will be employed (46). Using unsupervised or semi-supervised algorithms, the microbial community structure will be analyzed to identify species that contribute the most informative signals and downweight those containing redundant information, thereby eliminating bias in the resulting network. First, topological measures (e.g., centrality indices) will be incorporated as features in classification and regression problems. This approach will allow the model to be sensitive both to the individual variability of each variable and to its relationships with all others (i.e., other microorganisms). Second, a network will be constructed without assuming linear relationships between microbial taxa or applying standard regularization techniques. Ensemble methods (e.g., Random Forest, XGBoost, or AdaBoost) will be used to infer network connections. These methods enable the prediction of each variable based on all remaining n–1 variables by iteratively looping through them. The result is a matrix quantifying the importance of each variable in predicting all others. Additionally, adjusted R^2^ values will be calculated to estimate how predictable each node (e.g., microbial taxon) is when the values of all its neighboring nodes such as other microbial taxa, dietary variables, exercise, clinical data, LPS, or butyrate are known. This allows us to evaluate the conditional probability of change for each node given the state of its neighbors. The networks will be visualized using the qgraph R package (47) and the Fruchterman–Reingold algorithm (48) which positions nodes with stronger or more numerous connections closer together, while placing those with lower centrality toward the periphery. To assess network stability, we will follow the protocol of Costenbader and Valente (49) Stability indices will be computed for all networks to ensure the robustness of results within the dataset. Cohesion metrics (50) will also be estimated as individual indicators of community complexity, evaluating microbial interconnection. Since high cohesion may indicate that many taxa respond simultaneously to external forces, this metric is a promising candidate for correlating microbial network structure with external parameters.

### 2.20 Supplement design

Patients will be clustered into distinct groups based on the similarity of their baseline gut microbiota composition, clinical markers, and dietary patterns. Dimensionality reduction techniques, e.g., principal component analysis (PCA) or uniform manifold approximation and projection (UMAP) will be applied to reduce noise and identify key features driving interindividual variability. Subsequently, unsupervised clustering algorithms (e.g., k-means, hierarchical clustering, or model-based clustering) will be employed to classify patients into homogeneous subgroups. These patient clusters will serve as the basis for subsequent supplement design, analyses of intervention response and biomarker discovery. The dietary supplement will be formulated by selecting two or three food groups or food components that meet the highest number of predefined criteria. The selection criteria will be: (1) components positively associated with, or predictive of, a greater number of protective variables or markers of healthier status in Alzheimer’s patients; (2) components negatively associated with, or predictive of, a greater number of disease-related markers.

The rationale for designing a supplement rather than recommending dietary modifications is to minimize disruption of habitual dietary patterns, thereby maximizing adherence to the intervention and reducing caregiver burden. In addition to supplement formulation, dietary recommendations may be provided to reduce or eliminate food components associated with unfavorable biomarkers (e.g., predictors of elevated LPS levels or other parameters of disease progression). If lack of physical activity is identified as a relevant factor, tailored recommendations will also be considered according to the patients’ physical condition.

The selection of supplement ingredients will be based on analysis of patients’ dietary intake to ensure that nutrient levels do not exceed recommended intakes for the study population and that established diet quality indices are maintained. Individual dietary preferences and restrictions will also be taken into account. Food components will preferentially be included in powdered form to facilitate integration into common foods (e.g., soups, yoghurts, purees), while vitamins and minerals, if selected, will be administered as liquid or pill formulations. Dosage will be determined according to dietary reference values and available scientific literature supporting the beneficial health effects of the selected components.

### 2.21 Statistical analysis

The analysis of the obtained data will be carried out using the statistical software SPSS version 29.0. The mean and standard deviation, or the median and interquartile range, will be used to describe each parameter, depending on the data distribution. The assumption of normality will be assessed using the Shapiro–Wilk test. All continuous outcomes will be analyzed using linear models adjusting for the baseline value of the outcome and a set of covariates: age, sex, education level, comorbidity burden, concomitant medication, physical activity level, nutritional status, and perceived stress. Study site will be included as a fixed effect. For outcomes with repeated measures (e.g., pre/post within participants), we will use linear mixed-effects models with a random intercept for participant and treatment group (personalized supplement vs. control) as the main fixed effect. Where a single post-intervention endpoint is analyzed, an ANCOVA (post value as dependent variable; treatment as factor; baseline outcome and covariates as regressors) will be used. An intention-to-treat (ITT) approach will be employed to evaluate the effectiveness of the intervention. The ITT analysis will include all participants initially randomized, regardless of whether they completed the intervention as planned, withdrew, or deviated from the protocol. For gut microbiota analysis, RStudio (v. 1.4.1106, RStudio, 2022) will be used. The phyloseq R package (51) will be employed to compare different estimates of alpha diversity and to plot the relative abundance of microbial groups. The vegan R package (52) ^1^will be used to compute beta diversity, i.e., inter-sample diversity, visualized through Principal Coordinates Analysis (PCoA) based on the Bray–Curtis dissimilarity index. The significance and effect of treatments on the bacterial community (beta diversity) will be evaluated using PERMANOVA implemented in the vegan package. Differences will be considered statistically significant when the *p*-value is less than the alpha level (0.05). For microbiome, functional profiles and untargeted metabolomics, we will use MaAsLin2, a multivariable linear modeling framework designed for meta-omics data analysis (53) with appropriate normalization/transformations, adjusting for the same covariates listed above. Participant ID will be specified as a random effect for longitudinal analyses. For multiple comparisons, the Benjamini–Hochberg correction will be applied to control the false discovery rate. To address multiplicity, two complementary approaches will be applied depending on the type of outcomes. For a small number of primary or key secondary outcomes (e.g., plasma LPS, total SCFAs), we will apply the Bonferroni correction. For high-dimensional exploratory domains (microbiota taxonomic and functional profiles, untargeted metabolomics, and dietary intake variables), *p*-values will be adjusted using the Benjamini–Hochberg procedure to control the false discovery rate (FDR).

### 2.22 Ethics, samples destination, and confidentiality

This protocol has been approved by the Ethics Committees of Hospital Clínico Universitario Virgen de la Arrixaca (HCUVA) and Hospital Universitario Puerta de Hierro (HUPHM), under registration numbers 2023-5-9-HCUVA and PI 216/24, respectively. Except in emergency situations, no changes or deviations from the protocol will be permitted without documented approval. The Clinical Research Ethics Committee will be informed of any potential changes and must provide written approval for any modification or deviation that could increase risk to participants and/or adversely affect participants’ rights or the validity of the research. This requirement does not apply to changes made to reduce discomfort or avoid risk to participants, nor to changes affecting the administrative aspects of the study. The study will be conducted in accordance with the principles of Good Clinical Practice and the ethical standards established by the Declaration of Helsinki, as incorporated into current legislation governing clinical research. Informed consent will be obtained from all participants. All data collected in the study, both for inclusion in the subject’s clinical history and for other study documentation, will be stored at HCUVA or Universidad Complutense de Madrid (UCM), either in paper or digital format. Study data will be coded using a numerical identifier, and only the principal investigator (PI) and authorized collaborators will be able to link the data to individual participants and their medical records. Access to subject information will be restricted to the study physician and authorized members of the research team. The investigator and the study center will guarantee direct access to data or source documents for authorized personnel when required. Microbiota and metabolomic data will be made available in public repositories to promote transparency and support future research. Biological samples obtained in the study will become part of the biobank collections of either the IMIB-Arrixaca Biobank—registered in the National Biobank Registry of the Carlos III Health Institute under reference number PT17/0015/0038—or the Biobank of Hospital Universitario Puerta de Hierro (reference number B0000920), in accordance with the regulations established by the National Registry of Biobanks for Biomedical Research. Samples will be stored in research laboratories at UCM for scientific purposes. Participants may request the withdrawal of their consent at any time. The PI will ensure the anonymity and confidentiality of all participants. All documents will be maintained under strict confidentiality in accordance with national laws on the Protection of Personal Data and the Guarantee of Digital Rights.

### 2.23 Dissemination

The results of the project will be disseminated through scientific, healthcare, and public communication channels. From a scientific perspective, the research findings will be published in peer-reviewed articles in high-impact international journals and presented at both national and international conferences. Additionally, seminars will be held in public hospitals to share knowledge with healthcare professionals. Informative sessions will also be offered to individuals affected by AD, highlighting the role of diet in managing the condition. For public outreach, the research group will participate in events such as public seminars, “Science Week,” and the “European Researchers’ Night,” engaging with a wider audience. Media dissemination will include press releases and television appearances. Project updates will be provided through our website^2^, and we will organize open-access meetings at the UCM to share information on microbiota, diet, and healthy lifestyle. These events will also be broadcast via a dedicated YouTube channel. Upon project completion, national neurology specialists and patient associations will be contacted to communicate the project’s outcomes and directly transfer knowledge to patients. To be listed as a co-author in the resulting scientific publications, individuals must meet the criteria set by the International Committee of Medical Journal Editors (ICMJE) for the conduct, reporting, editing, and publication of scholarly work in medical journals. According to ICMJE guidelines, authors must have made substantial contributions to the work (e.g., conception, design, data acquisition, analysis, or interpretation), participated in drafting or critically revising the manuscript, approved the final version, and agreed to be accountable for all aspects of the work.

## 3 Discussion

The gut–brain axis, a complex bidirectional communication network between the gastrointestinal tract and the brain, plays a critical role in maintaining homeostasis and influencing brain function and behavior (2). Recent research has highlighted the potential role of gut microbiota on neurodegenerative diseases, particularly AD (54, 55). Dysbiosis of the gut microbiota has been linked to increased inflammation, altered immune responses, and the production of neurotoxic metabolites, all of which may exacerbate the pathogenesis of AD (54). This protocol will enable future investigation of whether modulating the gut microbiota through diet, probiotics, or other interventions (4, 12, 13), there is potential to influence the gut–brain axis and, consequently, the progression of AD, offering promising approaches for the future treatment, and potentially prevention strategies (55). This study presents an interdisciplinary approach and innovative methodologies to investigate the gut microbiota in patients with AD and to design personalized dietary supplements. Ultimately, the study aims to evaluate the effects of these supplements on gut microbiome profiles and the metabolome, with the goal of modulating microorganisms and metabolites associated AD offer limited symptomatic relief, underscoring the urgent need for alternative, non-invasive approaches that target the underlying pathophysiology (56, 57). The personalized nutritional strategy evaluated in this pilot interventional trial may provide insights into mechanisms, that could be relevant for future disease-modifying strategies in AD. Integrating gut microbiome analysis into clinical care for AD could support the development of more personalized prevention and treatment strategies (54–56).

The knowledge generated by this project could also serve as the basis for the development of new products in the food and pharmaceutical industries, where the analysis methodology could be applied to customize products based on specific consumer or patient characteristics. However, several limitations of the present study should be acknowledged. First, the reliance on self-reported dietary and lifestyle data introduces potential biases, including recall and social desirability bias. Second, adherence to the personalized supplement may be challenging, especially among individuals with AD, who may resist changes to their routine. Third, the use of caregivers as the healthy control group instead of community-based controls. Although this choice increases feasibility and facilitates adherence to study procedures, it may introduce potential biases related to caregiver stress, shared environment, and lifestyle similarities with patients. Fourth, potential issues may arise related to supplement formulation, its stability, or potential interactions with medications, all of which will need to be addressed during study implementation. Fifth, although the study aims to consider multiple factors (e.g., individual characteristics, microbiota, and diet), the influence of unaccounted confounding variables may affect the study outcomes. Sixth, the relatively short intervention period of 3 months limits the ability to evaluate long-term outcomes, including prevention or disease modification. Finally, despite biomarker confirmation, the AD population remains heterogeneous, which may influence microbiota profiles and intervention responses.

To address these limitations, several mitigation strategies have been incorporated into the study design. To minimize bias from self-reported data, standardized questionnaires will be used together with dietary diaries, and whenever possible, information will be cross-checked with caregivers. Adherence to supplementation will be promoted through clear instructions, weekly phone calls, and monitoring with intake diaries and returned supplement containers. The use of caregivers as the control group, while potentially introducing bias, ensures feasibility and compliance; these factors will be systematically documented and accounted for in the statistical analysis. Regarding supplement formulation, development will involve nutrition and food technology experts to ensure palatability, stability, and safety, with particular attention to possible interactions with common medications. Potential confounders will be carefully recorded and adjusted in multivariate analyses. Although the 3-month intervention limits evaluation of long-term outcomes, the study is designed as a feasibility trial to provide preliminary data and guide larger, longer studies. Finally, heterogeneity within the AD population will be considered by stratifying analyses according to relevant clinical and microbiota variables, thereby maximizing the interpretability and generalizability of the results.

In summary, this protocol describes the design and methodology of an exploratory study evaluating the gut–brain axis in biomarker-confirmed AD. While not intended to provide definitive outcomes, it will generate preliminary data on feasibility, adherence, and biological signals, thereby laying the groundwork for future prevention- and treatment-oriented research.

## Footnotes

^1^ https://github.com/vegandevs/vegan/

^2^ https://www.masmicrobiota.es/
